# Longitudinal and quantitative fecal shedding dynamics of SARS-CoV-2, pepper mild mottle virus, and crAssphage

**DOI:** 10.1128/msphere.00132-23

**Published:** 2023-06-20

**Authors:** Peter J. Arts, J. Daniel Kelly, Claire M. Midgley, Khamal Anglin, Scott Lu, Glen R. Abedi, Raul Andino, Kevin M. Bakker, Bryon Banman, Alexandria B. Boehm, Melissa Briggs-Hagen, Andrew F. Brouwer, Michelle C. Davidson, Marisa C. Eisenberg, Miguel Garcia-Knight, Sterling Knight, Michael J. Peluso, Jesus Pineda-Ramirez, Ruth Diaz Sanchez, Sharon Saydah, Michel Tassetto, Jeffrey N. Martin, Krista R. Wigginton

**Affiliations:** 1 Department of Civil and Environmental Engineering, University of Michigan, Ann Arbor, Michigan, USA; 2 Department of Epidemiology and Biostatistics, University of California, San Francisco, California, USA; 3 Institute for Global Health Sciences, University of California, San Francisco, California, USA; 4 Division of Hospital Medicine, UCSF, San Francisco, California, USA; 5 F.I. Proctor Foundation, University of California, San Francisco, California, USA; 6 National Center for Immunizations and Respiratory Diseases, Centers for Disease Control and Prevention, Atlanta, Georgia, USA; 7 Department of Microbiology and Immunology, UCSF, San Francisco, California, USA; 8 Department of Epidemiology, University of Michigan, Ann Arbor, Michigan, USA; 9 Department of Civil & Environmental Engineering, Stanford University, Stanford, California, USA; 10 School of Medicine, University of California, San Francisco, California, USA; 11 Division of HIV, Infectious Disease, and Global Medicine, UCSF, San Francisco, California, USA; Johns Hopkins Bloomberg School of Public Health, Baltimore, Maryland, USA

**Keywords:** SARS-CoV-2, stool, fecal shedding, PMMoV, crAssphage

## Abstract

Wastewater-based epidemiology (WBE) emerged during the coronavirus disease 2019 (COVID-19) pandemic as a scalable and broadly applicable method for community-level monitoring of infectious disease burden. The lack of high-resolution fecal shedding data for severe acute respiratory syndrome coronavirus 2 (SARS-CoV-2) limits our ability to link WBE measurements to disease burden. In this study, we present longitudinal, quantitative fecal shedding data for SARS-CoV-2 RNA, as well as for the commonly used fecal indicators pepper mild mottle virus (PMMoV) RNA and crAss-like phage (crAssphage) DNA. The shedding trajectories from 48 SARS-CoV-2-infected individuals suggest a highly individualized, dynamic course of SARS-CoV-2 RNA fecal shedding. Of the individuals that provided at least three stool samples spanning more than 14 days, 77% had one or more samples that tested positive for SARS-CoV-2 RNA. We detected PMMoV RNA in at least one sample from all individuals and in 96% (352/367) of samples overall. CrAssphage DNA was detected in at least one sample from 80% (38/48) of individuals and was detected in 48% (179/371) of all samples. The geometric mean concentrations of PMMoV and crAssphage in stool across all individuals were 8.7 × 10^4^ and 1.4 × 10^4^ gene copies/milligram-dry weight, respectively, and crAssphage shedding was more consistent for individuals than PMMoV shedding. These results provide us with a missing link needed to connect laboratory WBE results with mechanistic models, and this will aid in more accurate estimates of COVID-19 burden in sewersheds. Additionally, the PMMoV and crAssphage data are critical for evaluating their utility as fecal strength normalizing measures and for source-tracking applications.

**IMPORTANCE:**

This research represents a critical step in the advancement of wastewater monitoring for public health. To date, mechanistic materials balance modeling of wastewater-based epidemiology has relied on SARS-CoV-2 fecal shedding estimates from small-scale clinical reports or meta-analyses of research using a wide range of analytical methodologies. Additionally, previous SARS-CoV-2 fecal shedding data have not contained sufficient methodological information for building accurate materials balance models. Like SARS-CoV-2, fecal shedding of PMMoV and crAssphage has been understudied to date. The data presented here provide externally valid and longitudinal fecal shedding data for SARS-CoV-2, PMMoV, and crAssphage which can be directly applied to WBE models and ultimately increase the utility of WBE.

## INTRODUCTION

Severe acute respiratory syndrome coronavirus 2 (SARS-CoV-2) RNA is commonly shed in the feces of individuals infected by the virus (1 - 3). This has led to the widespread adoption of wastewater-based epidemiology (WBE) for tracking coronavirus disease 2019 (COVID-19) trends in communities. SARS-CoV-2 RNA concentrations in wastewater correlate with measures of COVID-19 incidence (4 - 8), hospitalizations (6, 9, 10), and deaths (8, 11). Although these correlations provide proxies for the relative levels of transmission in a sewershed over time, a mechanistic link between fecal shedding of SARS-CoV-2 RNA and wastewater monitoring data would strengthen the utility of WBE methods. For example, epidemiological models estimating total infections in a community (i.e., prevalence) (12, 13) or effective reproductive number (Re) (14) rely on the integration of fecal shedding and wastewater transport models. Establishing a link between epidemiological models and fecal shedding would also aid in identifying desirable sewershed sizes (15), choosing sampling frequencies (16), knowing when and how to normalize measurements (17), and probing the differences sometimes observed between wastewater and clinical trends (8, 10, 18).

WBE models require quantitative and longitudinal fecal shedding data so that the RNA sequences shed by individuals are accurately integrated into bulk wastewater concentrations. To date, there have been numerous published studies on SARS-CoV-2 RNA presence and abundance in stool, including several reviews (1 - 19 - 25 - 25). These important studies demonstrated that SARS-CoV-2 RNA is shed in feces; however, most of the data in these studies lack the external validity necessary to generate accurate WBE models. In other words, they do not include sufficient methodological and sample information for the data to be directly compared and integrated with other studies. For example, several studies have not specified the precise times that samples were collected in the infection (e.g., days after initial symptom onset). Quantitative data by quantitative PCR (qPCR) have often been reported in terms of cycle threshold (Ct) rather than absolute abundance or have not included methodological details that build confidence in the data (e.g., data recommended by MIQE guidelines such as method detection limits and negative controls) (26, 27). Additionally, many studies have either not specified the amount of stool analyzed or reported the stool volume analyzed rather than mass analyzed. No study to date has reported SARS-CoV-2 concentrations on a dry mass basis. Dry mass concentrations lead to more accurate estimates of genome copies shed per day as dry stool production rates vary less than wet stool production rates (28). These missing details in fecal shedding data have limited the epidemiological interpretation of wastewater data through modeling (12 - 14).

CrAss-like phage (crAssphage) and pepper mild mottle virus (PMMoV) are viral indicators of human fecal contamination and are often measured alongside SARS-CoV-2 nucleic acid concentrations in wastewater samples (4, 29). The measured pathogen viral nucleic acid concentrations are normalized by the crAssphage or PMMoV viral nucleic acid concentrations to account for differences in wastewater fecal strength and viral nucleic acid recovery. To date, there is very little quantitative data available on the levels and temporal trends of these biomarkers in feces. These quantitative data are important for mechanistically linking normalized wastewater pathogen measurements with community disease burdens and for identifying the scenarios in which biomarker normalization is appropriate. Models that have incorporated PMMoV fecal shedding data to estimate the fecal strength of wastewater have relied on very limited data on PMMoV fecal shedding (12, 29). A quantitative understanding of biomarker shedding by individuals would help inform the scenarios that biomarkers serve as accurate, representative measures of community fecal loads.

In this study, we present externally valid quantitative fecal shedding trajectories of SARS-CoV-2 RNA and commonly used biomarkers PMMoV and crAssphage from 48 individuals who tested positive for COVID-19. The results show a highly individualized course of SARS-CoV-2 shedding over the first 30 days after initial onset of symptoms (ASO). Shedding of PMMoV and crAssphage was also highly variable between individuals over the same sampling period, and the two indicators exhibited distinct shedding patterns. Together, these results provide critical data for advancing the utility of WBE as a public health tool.

## RESULTS

### Cohort description

In total, 48 individuals provided stool samples for this study. Four of the 48 individuals did not experience symptoms of acute COVID-19. Of those with symptoms, the earliest stool samples were collected at 3 days pre-symptom onset and the latest samples were collected on day 28 after initial acute symptom onset. Three hundred eighty-two samples were collected from 26 index individuals and 22 household contacts who became infected between September 2020 and April 2021. Of the individuals, 58% were females and 42% were males. The age breakdown was as follows: 17% 0–17 years old, 67% 18–55 years old, and 17% above 55 years old (additional information in Table S1). The self-reported ethnicity/race breakdown was as follows: 38% White (18/48), 27% Asian (13/48), 25% Hispanic/Latino (12/48), 4% Black/African American (2/48), 2% American Indian or Alaska Native (1/48), 2% Native Hawaiian or Pacific Islander (1/48), and 2% declined to answer(1/48). The cohort also contained four individuals who were fully vaccinated for SARS-CoV-2.

### SARS-CoV-2 fecal shedding

#### Analysis summary

**Fig 1:**
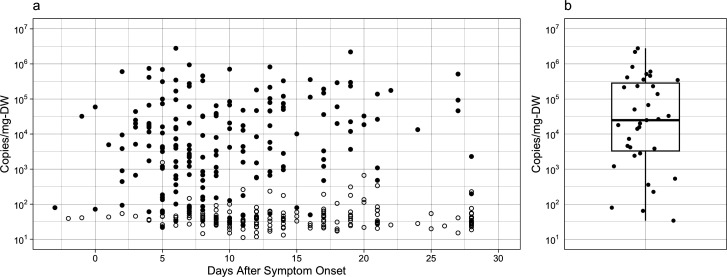
SARS-CoV-2 RNA fecal shedding data. (a) Longitudinal plot of all SARS-CoV-2 N gene measurements. Empty symbols represent sample measurements below the LOB. (b) Boxplot and individual data points summarizing the peak fecal shedding magnitude for each individual with at least one sample above the LOB.

Quantitative measurements of SARS-CoV-2 RNA were conducted on 382 samples from 48 individuals (Fig. 1a). The concentration of fecally shed SARS-CoV-2 N gene was plotted against the time since symptom onset. Data for the four asymptomatic individuals were plotted in reference to the symptom onset of the identified index case. The median number of samples collected per individual was 9, with a range of 1–15 samples per individual. Samples were collected between −3 and 28 day ASO. Assays targeting the viral genes N and ORF1a exhibited a log-linear correlation (*r*^2^ = 0.85; Fig. S1A); therefore, all subsequent analyses of SARS-CoV-2 RNA shedding used only the quantitative N gene data. Bovine coronavirus-modified live-virus vaccine (BCoV) was spiked in each sample prior to extraction and quantified to ensure sufficient viral nucleic acid recovery through processing. Three samples were ultimately excluded from analysis due to BCoV recoveries under 50%, and thus our final data set consisted of 379 samples. The limit of blank (LOB) for our SARS-CoV-2 RNA concentrations, determined as the upper 95% confidence limit of the negative extraction control (30), ranged from 11.2 to 1,550 gene copies/milligram-dry weight (gc/mg-dry weight), with differences between analytical runs and between samples due to varying levels of background signals in our negative extraction controls and differences in the sample solid content. The LOB was used as the threshold of positivity for digital droplet PCR (ddPCR) to account for run-to-run changes in background levels. The method of calculation for the LOB is included in the ddPCR methods section.

#### Shedding prevalence among population

In summary, 51% (193/379) of all collected samples resulted in SARS-CoV-2 N gene measurements above the LOB, and 73% (35/48) of the individuals contributed at least one sample above the LOB. Of the remaining 27% of all individuals who had no stool samples positive for SARS-CoV-2 RNA, three individuals (4542, 4567, and 4571) provided fewer than three samples over the course of the sampling period, limiting the coverage of a potential period of fecal shedding (Fig. S2). Here, if we only include individuals who contributed at least three samples spanning at least 15 days between the earliest and latest collected sample, the proportion of participants without positive measurements decreased to 23% (7/31). It remains possible that these individuals excreted SARS-CoV-2 RNA on days when samples were not collected or at levels that were below our LOB. Three of the four vaccinated individuals included in this cohort had measurable SARS-CoV-2 RNA in their stool at some point in the sampling period (Supplementary Data, Demographics).

#### Shedding prevalence over time

**Fig 2:**
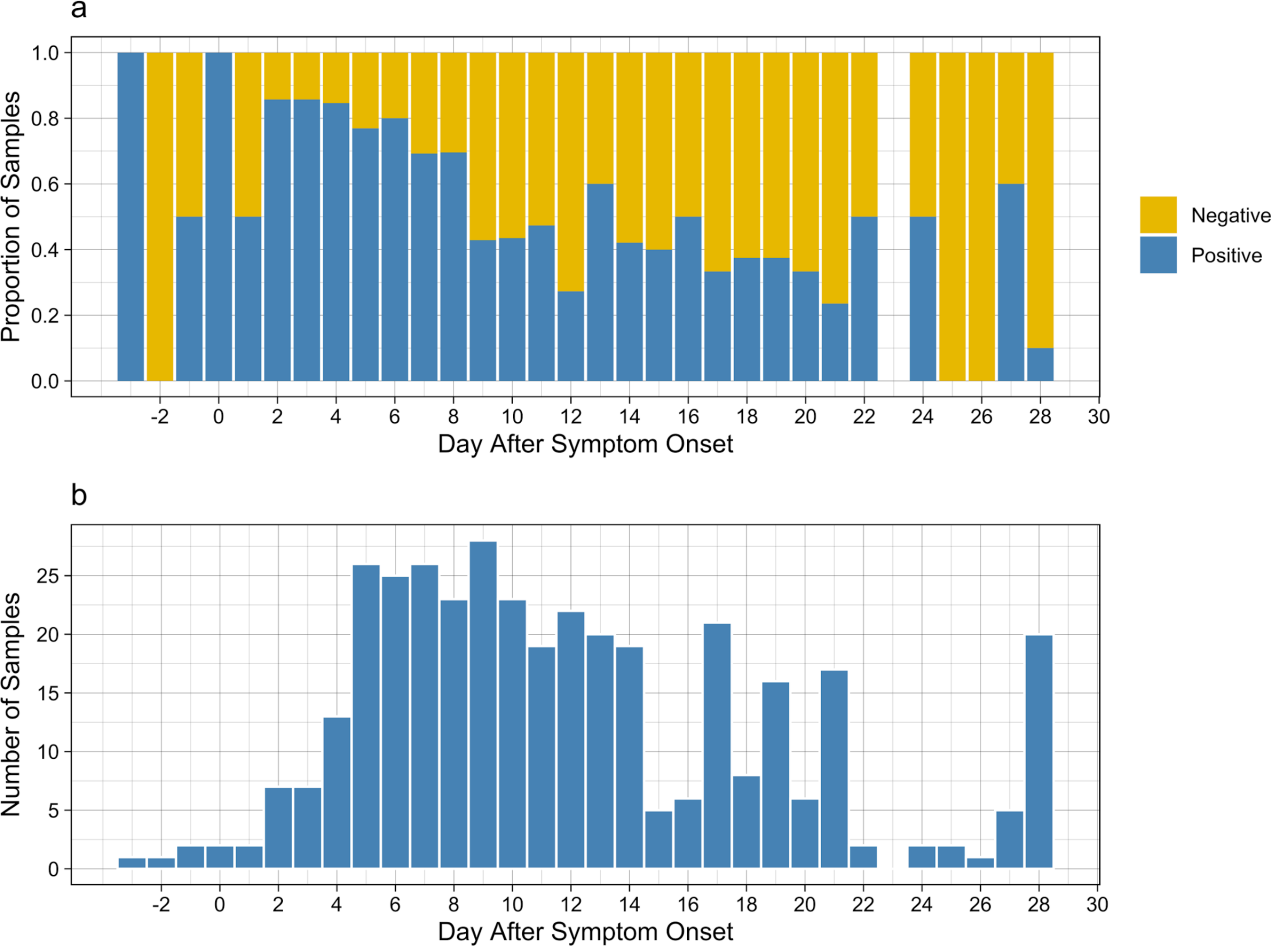
(a) The proportion of stool samples on each day after symptom onset where SARS-CoV-2 N gene was measured above the LOB. (b) A histogram showing the number of samples measured on each day after symptom onset.

To understand the prevalence of fecal shedding on each day after the initial acute onset of symptoms in the studied population, we calculated the proportion of samples collected for a specific day that were positive for SARS-CoV-2 RNA (Fig. 2a). Prior to symptom onset, as well as on the first day ASO, the number of samples per day are limited (Fig. 2b), making it difficult to draw conclusions about the prevalence of fecal shedding during this period. We note that two of the four samples collected pre-symptom onset were positive for SARS-CoV-2 RNA, corresponding to two of three household contacts that contributed pre-symptomatic samples. These results provide important evidence of pre-symptomatic fecal shedding of SARS-CoV-2 RNA. Our sample set includes more samples per day from day 2 ASO through day 20 ASO and thus presents a clearer picture of shedding prevalence during that time. The prevalence of fecal shedding peaks at 86% on days 2 and 3 ASO and then this percentage decreases until reaching 10% on day 28 ASO.

A mixed-effect logistic regression was applied to the shedding prevalence data for each day to test the fixed effects of day ASO and sex, with the influence of each individual as the random effect (Table S3). Our results demonstrate a decrease in the probability of SARS-CoV-2 fecal shedding with increasing days ASO with an odds ratio of 0.6 (log_10_ estimate = −0.23). The effect of sex on the observed fecal shedding was not statistically significant. Demographics such as age, symptomaticity, vaccination status, and ethnicity were excluded due to insufficient sample sizes across groups to provide significant information.

#### Quantitative measurements

The quantitative reverse transcriptase (RT)-ddPCR measurements coupled with stool percent solids’ measurements provide externally valid SARS-CoV-2 RNA absolute abundance data. The geometric mean of all measurements above the LOB was 5.3 × 10^3^ gc/mg-dry weight, the geometric standard deviation was 18 gc/mg-dry weight, and the median was 4.8 × 10^3^ gc/mg-dry weight. The maximum shedding value observed across all 379 samples was 2.8 × 10^6^ gc/mg-dry weight, and the minimum positive shedding value observed was 22 gc/mg-dry weight. The peak SARS-CoV-2 N gene concentration for individuals with sufficient sample coverage varied over approximately six orders of magnitude (Fig. 1b). The maximum and minimum measured peak values for individuals were 2.8 × 10^6^ gc/mg-dry weight and 34 gc/mg-dry weight, respectively. The median peak shedding value was 1.9 × 10^4^ gc/mg-dry weight, the geometric mean was 1.3×10^4^ gc/mg-dry weight, and the geometric standard deviation was 18 gc/mg-dry weight.

### PMMoV and CrAssphage fecal shedding

#### Analysis summary

**Fig 3:**
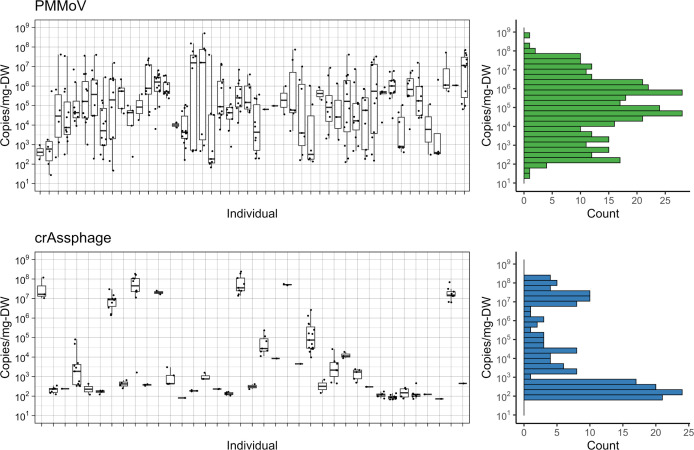
Above LOB or LOD shedding measurements and histogram of PMMoV (top) and crAssphage (bottom) for all samples in this study, as measured by qPCR (crAssphage) or RT-ddPCR (PMMoV). In the box plots (left), each box summarizes all measurements available for an individual with the bar indicating the median shedding value. The histograms (right), summarize the distribution of shedding magnitude for the entire set of above detection limit measurements.

PMMoV and crAssphage nucleic acids were measured in each stool sample to observe the biological variability of commonly used fecal biomarkers between individuals and within individuals over the sampling period (Fig. 3). PMMoV RNA was measured by ddPCR on the same day as the SARS-CoV-2 and BCoV RNA measurements, and the three samples with BCoV recoveries less than 50% were excluded from the data analyses. An additional 12 samples from four individuals (4512, 4514, 4545, 4576) were excluded from PMMoV data analysis due to an insufficient separation between background and positive droplets, possibly due to degeneracies between the designed assay target and the sample sequences.

#### Pepper mild mottle virus (PMMoV)

For PMMoV, 96% (352/367) of the measured samples had values above the LOB (mean LOB = 400 gc/mg-dry weight) and 100% (48/48) of individuals had at least one sample above the LOB. The median concentration of PMMoV RNA in feces was 1.0 × 10^5^ gc/mg-dry weight, and the maximum concentration observed was 5.0 × 10^8^ gc/mg-dry weight (Table 1). PMMoV RNA measurements varied highly between individuals and over the sampling period (Fig. S3). Some participants had consistent concentrations of PMMoV across many stool samples, whereas others had concentrations that varied by orders of magnitude between samples (Fig. 3). The median range of measurable PMMoV RNA in individuals with at least three samples was 7.0 × 10^6^ gc/mg-dry weight.

**TABLE 1:** Summary of PMMoV and crAssphage nucleic acid shedding data from the samples measured in this study

	PMMoV (gc/mg-dw)	CrAssphage (gc/mg-dw)
Geometric mean	8.7 × 10^4^	1.4 × 10^4^
Geometric SD	37.1	148
Median	1.0 × 10^5^	1.9 × 10^3^
Fraction of samples positive	0.96 (352/367)	0.48 (179/371)
Fraction of individuals positive	1.00 (48/48)	0.79 (38/48)

#### CrAss-like phage (crAssphage)

CrAssphage DNA shedding was observed less frequently than PMMoV RNA shedding in the study participants (Fig. S3 and S4). In summary, 48% (179/371) of all samples were above the crAssphage assay limit of detection (LOD) (mean LOD = 25 gc/mg-dry weight) and 79% (38/48) of individuals had at least one sample above the LOD. For qPCR, an assay LOD was used as the threshold of positivity (see qPCR analysis section for details). Of the samples that were positive for crAssphage DNA, the maximum fecal concentration was 2.4 × 10^8^ gc/mg-dry weight, and the median concentration was 2.1 × 10^3^ gc/mg-dry weight (Table 1). CrAssphage DNA in samples from some individuals were consistently high, while others consistently shed much lower concentrations or no detectable crAssphage DNA (Fig. 3 and Fig. S4). CrAssphage DNA fecal shedding was more consistent for individuals over time than PMMoV RNA shedding (Fig. S4). The median range of shedding values within an individual was 370 gc/mg-dry weight for crAssphage and 7.0 × 10^6^ gc/mg-dry weight for PMMoV. CrAssphage DNA levels varied less than three orders of magnitude for each individual, whereas PMMoV RNA levels varied more than three orders of magnitude for nearly all individuals (44/45). Interestingly, crAssphage concentrations in feces exhibited a bimodal distribution with most positive individuals shedding below 10^4^ gc/mg-dry weight (25/38) and a smaller fraction of individuals shedding above 10^6^ gc/mg-dry weight (7/38) (Fig. 3). An even smaller fraction of individuals exhibited intermediate magnitudes of shedding between 10^4^ and 10^6^ gc/mg-dry weight (6/38).

A mixed-effect linear regression was applied to test if differences in biomarker fecal shedding levels were observed based on sex, SARS-CoV-2 fecal shedding levels, and the fecal shedding of the other biomarker. In the case of both crAssphage and PMMoV, none of the fixed effects had a statistically significant effect on the biomarker shedding levels (Table S4). The random effects associated with PMMoV shedding were less variable than those associated with crAssphage, with standard deviations of 0.82 and 2.2 log_10_ gc/mg-dry weight, respectively.

## DISCUSSION

Here, we report quantitative and externally valid data on the fecal shedding of several targets of interest for wastewater-based epidemiology. This study features a large sample size and good sampling coverage over approximately 30 days. The resulting novel data set provides important information about the presence, magnitudes, and trends of viral nucleic acid fecal shedding among individuals. Included in our data set is evidence of SARS-CoV-2 fecal shedding in pre-symptomatic and vaccinated individuals.

There has been a sustained interest in the fraction of infected individuals that shed SARS-CoV-2 in feces, and we observed that approximately 22% of individuals who provided multiple samples did not shed SARS-CoV-2 in their feces up to day 28 ASO. A recent meta-analysis of fecal shedding of 38 individuals saw 52% of individuals did not shed SARS-CoV-2 RNA in feces; inspection of the included studies, however, revealed that the median number of samples collected per individual was 2 (3). Presumably, if more samples were collected from each study participant, this percentage may decrease. The observed 22%, reported in our study, included only those individuals with more than three samples collected over >15 days of their infection; it is likely that increasing the resolution of samples collected over the first 30 days of infection (e.g., daily) would result in an even lower percentage of individuals without positive SARS-CoV-2 samples.

Our high-resolution data and relatively large study population provided a unique description of the prevalence of shedding through the first 30 days ASO. Approximately 80% of samples collected within the first 5 days were positive for SARS-CoV-2, and this percentage dropped to 10% of samples at 28 days ASO. Natarajan and colleagues (2) collected samples from a large number of individuals (120) and collected three samples per individual over the 30 days ASO. They found that approximately 75% of individuals were shedding SARS-CoV-2 RNA in samples collected between days 0 and 7 and less than 25% of individuals were shedding SARS-CoV-2 RNA in samples collected between days 22 and 35 ASO. Although our data set ends at 28 days ASO, Natarajan et al. also observed shedding in a small fraction of individuals up to 288 days ASO.

The quantitative SARS-CoV-2 data presented here highlights the large variability in fecal shedding trajectories and magnitudes of SARS-CoV-2 between individuals. For example, of those who shed SARS-CoV-2 RNA at some point during their infection, peak shedding varied from as soon as 2 days ASO to 27 days sampling period (Fig. S2) and peak values span approximately four orders of magnitude (Fig. 1b). A study by Wölfel and colleagues (1) contained the most comprehensive fecal shedding trajectories prior to our study, with a set that included 60 stool samples from nine participants with mild-to-moderate COVID-19. Wölfel et al. also observed peak shedding values that ranged from four orders of magnitude. They reported RNA copies on a wet stool mass basis and did not report the percent solids for their samples; therefore, we made assumptions about the solid contents of their samples to directly compare our quantitative data (see Fig. S6 and S8 in the supplementary information). Overall, the SARS-CoV-2 quantities in our study are higher than those reported in the Wölfel study (Fig. S8). Specifically, the Wölfel et al. maximum shedding value of 2.4 × 10^5^ gc/mg-dry weight was an order of magnitude lower than the maximum concentration observed in our sample set (2.8 × 10^6^ gc/mg-dry weight). Likewise, the geometric mean from Wölfel et al. (157 gc/mg-dry weight) was two orders of magnitude lower than our geometric mean (1.6×10^4^ gc/mg-dry weight). These large differences in peak and mean concentrations may be due to the larger number of individuals included in our study (48) compared to the Wölfel study (9).

Interestingly, some of the stool samples measured in our study that were positive for SARS-CoV-2 RNA at 25+ days ASO had relatively high concentrations (i.e., greater than 10^5^ gc/mg-dry weight). Although the quantities reported in the Natarajan study cannot be directly compared with other studies due to missing data on stool masses, the relative abundance between samples suggests that the maximum fecal concentrations at 25+ days after symptom onset are nearly as high as the maximum concentrations measured at 0–7 days after symptom onset. Combined, the data from our study and the study by Natarajan demonstrate that although fewer infected individuals are shedding SARS-CoV-2 in their feces by 28 days after symptom onset, some can be excreting high levels of SARS-CoV-2 RNA.

These data and observations on SARS-CoV-2 fecal shedding have particular value for advancing the field of WBE. For example, the pre-symptomatic shedding helps explain why wastewater measurements sometimes precede COVID-19 clinical cases (18). Likewise, observations of high levels of shedding weeks into an infection suggest that some individuals several weeks into their infection contribute substantially to measured wastewater signals. We anticipate that these data will be especially impactful for informing mechanistic models. Indeed, the need for high-quality fecal shedding data sets has been highlighted in published studies that use wastewater data in epidemiological models (12 - 14). The idiosyncrasies of the shedding trajectories highlight the potential complications of using models to directly predict epidemiological parameters such as community prevalence. Such attempts have often relied on static distributions rather than trajectories; however, recent work with polio and SARS-CoV-2 has demonstrated methods for incorporating time-varying shedding into mechanistic WBE models (10, 31). Using a static distribution for fecal shedding assumes a uniform likelihood of being at any stage in the shedding period. However, early in an outbreak, the majority of infected individuals are likely in the early stages of infection, while late in an outbreak, infected individuals are more likely a mix of early- and late-stage infections. We see that different individuals shed at dramatically different rates at different stages of the first 4 weeks after symptom onset. The impact of these patterns can be explored in the future by integrating our data into time-varying fecal shedding models of WBE systems.

This work also fills a critical research gap on the fecal shedding of two commonly used fecal indicator organisms, namely PMMOV and crAssphage. WBE studies routinely present pathogen nucleic acid concentrations on a per PMMoV or crAssphage nucleic acid concentration basis to normalize for differences in wastewater fecal strength and the analytical recovery of viral nucleic acids. Biomarker fecal concentrations have been applied in WBE models (12, 14), but the data have been limited by the number of individuals observed, the external validity of the measurements, or both. The PMMoV RNA and crAssphage DNA quantities presented here for 48 individuals over time therefore significantly improve available information on the absolute abundance and variability of these biomarkers in the stool.

We observed PMMoV in nearly all samples (96%) and in at least one sample from all individuals, with a median concentration of 1.0 × 10^5^ gc/mg-dry weight and a maximum concentration of 5.0 × 10^8^ gc/mg-dry weight. The limited previous studies on PMMoV RNA in the stool have detected it less frequently, with 40% of samples from five individuals detected by metagenomic techniques (32) and 67% of samples from nine individuals detected by PCR (33 ). Our PMMoV concentrations are within the range of three stool samples that were quantified in a previous study by RT-qPCR (33). That study reported concentrations of 2.3 × 10^4^, 3.6 × 10^6^, and 2.0 × 10^5^ gc/mg-stool. Assuming a 20% dry mass in their samples, the equivalent dry mass concentrations would be 4.6 × 10^3^, 7.3 × 10^5^, and 3.9 × 10^4^ gc/mg-dry weight, respectively.

CrAssphage DNA was detected in 48% of our samples and in at least one sample from 79% of all individuals. The maximum fecal concentration of crAssphage was on the same order of magnitude as the maximum PMMoV fecal concentration, namely 2.4 × 10^8^ gc/mg-dry weight; however, the median concentration of crAssphage, 2.1 × 10^3^ gc/mg-dry weight, was nearly two orders of magnitude lower than the median concentration of PMMoV. We identified only one previous study that reported crAssphage shedding quantities. That study reported their concentrations with respect to wet stool mass and collected only one sample from each individual (34). They observed 70% crAssphage shedding prevalence in 60 individuals infected with norovirus, 48% shedding prevalence in 96 healthy adults, and 69% shedding in 77 healthy children. They reported a wide range in crAssphage concentration, between 6.3 × 10^2^ and 2 × 10^10^ gc/g-stool. Other previous studies have documented crAssphage DNA in feces, but without quantification (35 - 37).

The contrasting distributions of crAssphage and PMMoV shedding could have implications for their use as a normalizing factor for WBE results. The presence of PMMoV RNA in stool is largely due to the consumption of pepper products (38), so the variability of PMMoV RNA fecal shedding likely relates to the range of diets between individuals and for individuals over time. These variations may complicate the practice of using PMMOV as a normalizing measure of fecal strength in some situations, such as for small sewersheds or for applications that assume similar PMMOV shedding between communities with different diets and in a single community over time. On the other hand, the bimodal fecal concentration distribution of crAssphage suggests that crAssphage DNA measurements may also not be a useful normalizing tool in some circumstances. Namely, large inconsistencies in the crAssphage concentration could occur depending on which individuals are contributing to the sample. These data are critical for understanding the utility of applying biomarker-based normalizing approaches to WBE, and we anticipate this biomarker shedding data will be used to assess the feasibility of such approaches in different contexts.

One of the most important aspects of the SARS-CoV-2, PMMoV, and crAssphage data sets presented here is that they are externally valid. In other words, the laboratory data were collected and reported in a way that makes the reported quantities useful beyond the context of this study. For example, we report absolute abundances of SARS-CoV-2 RNA. Relative abundance data, such as those reported as qPCR Ct are not able to be converted to precise absolute abundance measures without a standard curve calibrated for the methodological context. Likewise, we measured precise sample mass in our extractions and report our data on a per mass basis. Gene copy data reported per PCR reaction or nucleic acid extract volume without sample mass are not generalizable without making assumptions about sample collection. Finally, by reporting our results on a dry mass basis, we improve the ability to accurately estimate the quantity of SARS-CoV-2 RNA that is shed in an event or over a period of time. This is because the dry mass production of feces among populations is less variable than the wet mass production of feces (28). Consequently, the conversion of SARS-CoV-2 measurements in fecal samples to the SARS-CoV-2 generated in feces by an individual will carry less uncertainty when the fecal data are reported on a per dry mass basis.

We note that the identified limitations of the available SARS-CoV-2 fecal shedding literature are likely due to different priorities between fields. For WBE, accurate fecal shedding data that are reported as absolute abundance per dry fecal sample mass is critical for linking observations in wastewater with population epidemiological measures, such as infection prevalence or *R*_0_ values. In other applications, SARS-CoV-2 fecal shedding has been investigated to better define COVID-19 disease and help in the identification and treatment of participants. In these cases, measurements that identify the presence/absence of the target or the relative abundance of the target between samples, as opposed to externally valid absolute abundances, have been sufficient. Nonetheless, externally valid and accurate quantitative data will have the broadest utility and benefit a wider range of fields interested in fecal shedding. We therefore encourage future fecal shedding studies to pursue external validity of fecal shedding measurements and incorporate dry mass data.

There are several limitations to our data set as well as opportunities for future work. First, the subjects included in this study were from a relatively small geographic area–all were residents of the San Francisco Bay area. Additionally, the age and demographic distributions, including vaccination status, of the sample population were not large and diverse enough to identify demographic effects on the fecal shedding of SARS-CoV-2, PMMoV, and crAssphage nucleic acids. Furthermore, the samples measured in this study were collected between September 2020 and April 2021, prior to the emergence of the delta or omicron variants and we do not yet know how fecal shedding dynamics are affected by different variants. A limitation of the biomarker data is that these measurements were only made in individuals who had tested positive for SARS within 30 days and future work should quantify crAssphage and PMMoV shedding in healthy individuals. Despite these limitations, these quantitative data fill knowledge gaps on SARS-CoV-2 and viral biomarker fecal shedding and are critical for the advancement of WBE data interpretation. Our data can be directly compared and consolidated with future SARS-CoV-2 shedding studies, including those focused on the effects of demographics, vaccine status, and variants on fecal shedding.

## MATERIALS AND METHODS

### Cohort description and stool sample collection

Stool samples were collected by the FIND COVID project, a Centers for Disease Control and Prevention-funded cohort study of SARS-CoV-2 infectivity and viral transmission among households in the San Francisco Bay area (39, 40). Individuals with positive, provider-ordered PCR tests for SARS-CoV-2 RNA were eligible if they were not hospitalized and lived in close contact with at least one other individual. Household contacts of index cases were defined as anyone who spent at least one night in the household any time between 2 days before illness onset and index case enrollment and tested positive for SARS-CoV-2 after the exposure. Household contacts were ineligible if they had any previous history of SARS-CoV-2 infection or had suspected infection in the 14 days leading up to index case illness onset. Index cases and their household members were instructed to self-collect stool samples periodically over approximately 30 days after index case symptom onset. Specimens were collected in 50-mL conical stool sampling tubes before immediate storage at −20°C. Samples were transferred to −80°C within 1 week of collection where they were stored for up to 8 months. Samples were shipped from the University of San Francisco (UCSF) to the University of Michigan on dry ice, where they were immediately stored at −80°C prior to extraction and analysis. The study was reviewed by the UCSF Institutional Review Board (IRB) and given a designation of public health surveillance according to federal regulations as summarized in 45 CFR 46.102(d)(1)(2). Written informed consent was obtained from all participants. Analysis of deidentified patient samples was designated IRB exempt by the University of Michigan Institutional Review Board.

### Sample processing

Protocols for stool sample processing and extraction were adapted from previously published procedures for the analysis of SARS-CoV-2 RNA in fecal samples (41). Prior to analysis, stool samples were thawed on ice from −80°C. Between 10 and 25 mg of sample was added to a 2-mL screw cap tube with a rubber O-ring (Corning, CAT no. 430915). Next, 1,200 µL of DNA/RNA shield (Zymo, CAT no. R1100) and 0.5 g silica zirconia beads (BioSpec, CAT no. 11079105z) were added and the samples were homogenized using a BioSpec bead beater (BioSpec, CAT no. 1001). BCoV vaccine (Merck) was resuspended according to the manufacturer’s instructions and was added to the homogenate as a recovery control (1.5 µL/mL—DNA/RNA shield). BCoV was added to all samples as well as blanks at ~500 gc/µL. Recovery in each stool homogenate was calculated by dividing the concentration recovered in the sample by the concentration recovered in the DNA/RNA shield negative extraction control. Total nucleic acid was extracted with the Chemagic Viral DNA/RNA 300 kit H96 (Perkin Elmer no. CMG-1033-S) using the Chemagic 360 automated extraction platform (Perkin Elmer no. CMG-360). Samples were extracted in triplicate, along with triplicate samples of nuclease-free water as negative extraction controls. Extracts were further processed with Zymo PCR inhibitor removal kits (Zymo, CAT no. D6035), following manufacturer instructions. RNA samples were stored at 4°C prior to analysis.

The dry mass fraction for each sample was measured and is included in the supplementary information. In short, the sample dry mass fraction was measured by weighing a portion of the sample and placing it in a microcentrifuge tube with a hole pierced in the cap for venting. The tubes were placed in a heating block at 99°C for 24 hours before the sample was weighed again.

### ddPCR analysis

All ddPCR reactions were performed using the Advanced One-step RT-ddPCR Advanced kit for Probes (BioRad, Cat no. 1864022). Two different ddPCR approaches were used to quantify the targets of interest. At first, a duplex RT-ddPCR assay was used and RNA extracts were analyzed twice, once for SARS-CoV-2 N and ORF1a, and again for BCoV and PMMoV. This approach used separate channels for each target using the following concentrations of primers and probes: 2.7 µM of each primer and 0.75 µM of each probe. Later, a multiplexed RT-ddPCR assay to quantify all four targets in a single plate was implemented to be more resource efficient. Such a technique is described in the dMIQE guidelines (26) and was validated in our laboratory to produce comparable results from the same samples (Fig. S9). Primers and probes used in these assays were developed previously and have been used in published research (Table S1) (4). In short, 22-µL reactions contained 1.8 µM of SARS-CoV-2 N and ORF1a gene primers, 0.5 µM N 6-carboxyfluorescein (FAM) and ORF1a hexachlorofluorescin (HEX) hydrolysis probes, 0.9 µM PMMoV and BCoV primers, and 0.25 µM PMMoV (HEX) and BCoV (FAM) probes. All other reaction components were included at concentrations suggested by the manufacturer. Triplicate extraction replicates were plated for each sample, along with triplicate extraction negative controls and triplicate no template controls. Triplicate mixed-positive controls containing all four targets: (i) PMMoV gBlock gene fragment (IDT), (ii) extracted RNA from the BCoV vaccine, and (iii) SARS-CoV-2 gene fragment (NIST), were run on each plate. For each sample, extract dilutions of 1:10 and 1:100 were included to ensure optimal quantification and to identify inhibition effects. Analysis was performed using a BioRad ddPCR system (BioRad, Cat no. 1864100).

Thresholding was conducted with Bio-Rad QuantaSoft Analysis Pro Software (version 1.0). Only wells with >10,000 total droplets generated were included in the analysis. The LOB was calculated from the upper 95% confidence limit of the extraction negative control on each reaction plate (30). Reaction concentrations (gene copies/reaction) given by the instrument were converted to gene copies/milligram-dry weight using the mass of the sample extracted and the dry mass fraction of the sample, and a sample calculation is provided in the supplementary information (Equation S1). RT-ddPCR inhibition was observed in many of the measured samples, where the 1:100 extract dilutions ultimately resulted in higher target concentrations than the 1:10 dilutions. The influence of PCR inhibition was minimized by adopting the following procedure: when multiple dilutions of a sample provided a measurement above the detection limit, the highest dilution was selected. If none of the dilutions provided a measurement above the LOB, the LOB of the least diluted replicate was recorded as a non-detect. BCoV was measured as a positive recovery control and to ensure amplification and validate dilution series. Data were not used from samples where the BCoV recovery was less than 50%.

### qPCR analysis

A separate qPCR assay was used to quantify crAssphage DNA as adding in a fifth target for the ddPCR assay resulted in unreliable thresholding. Additionally, qPCR has a much larger single-reaction dynamic range that proved useful for the highly variable crAssphage DNA concentrations in the stool. Using a qPCR assay therefore eliminated the need of plating multiple dilutions per sample. The primers and probes used in this study were developed previously (42). Additional sequence information and cycling parameters are included in Table S2. PCR reactions were performed using the Luna Universal Probe qPCR master mix (New England Biolabs, Cat no. M3004), according to the manufacturer’s instructions. Ct data were converted to gene copies/reaction using a calibration curve consisting of eight dilutions, in duplicate, of a gBlock gene fragment (IDT) synthesized for the amplicon of interest. Dilutions of the standard were targeted to achieve a dynamic range of 1 gc/μL-rxn to 10^8^ gc/μL-rxn. The gBlock was quantified by duplicate measurements of three different dilutions using a ddPCR assay run concurrently with each qPCR run. The mean dilution-corrected concentration was then used to extrapolate across the standard curve. The *R*^2^ of the linear regression of the standard curve was always greater than 0.98, and the amplification efficiency was always greater than 87%. Triplicate negative extraction controls and no-template controls were included with every run and were considered acceptable if the concentrations were less than 1.5 cp/μL-rxn. The threshold of positivity for qPCR results was set as the Ct of the most dilute calibration curve standard that had a Ct lower than the negative extraction control.

### Statistical analysis

All statistical analyses were performed in R studio (version 4.1.2). To examine the dependency of SARS-CoV-2 fecal shedding prevalence to different variables, we used mixed-effect logistic regression. Logistic regressions were run using the “lme4” package with the glmer() function with the argument “family = ‘binomial’” (43). The formula used in this command was: SARS-CoV-2 shedding (T/F) ~ Day after symptom onset + Sex + (1 | individual identifier).

To investigate the dependency of the magnitude of crAssphage and PMMoV shedding, we used linear regression. This analysis was performed using the lmer() function from the lmer4 package. In this analysis, the formula we used was log10(biomarker_concentration) ~ Sex + SARS-CoV-2 shedding (T/F) + log10(alternative_biomarker_concentration) + (1 | individual identifier). The estimates and associated statistics associated with these results are included in Table S4. Individual differences were investigated as the random effect.

## SUPPLEMENTAL MATERIAL

Supplemental figures and tables msphere.00132-23-s0001.pdf Tables S1 to S4, Equation S1, and Fig. S1 to S12.

Supplemental Data msphere.00132-23-s0002.xlsx Demographic data for the study population, as well as the raw quantitative data collected during this study.

ASM does not own the copyrights to Supplemental Material that may be linked to, or accessed through, an article. The authors have granted ASM a non-exclusive, world-wide license to publish the Supplemental Material files. Please contact the corresponding author directly for reuse.
