# Supplementary material for: Longitudinal and quantitative fecal shedding dynamics of SARS-CoV-2, pepper mild mottle virus, and crAssphage
Source: mSphere. 2023 Jun 20;8(4):e00132-23. doi: 10.1128/msphere.00132-23 (PMC10506459; doi:10.1128/msphere.00132-23)
Supplement: Supplemental figures and tables — Tables S1 to S4, Equation S1, and Fig. S1 to S12. [file msphere.00132-23-s0001.pdf]

Supplementary Information

Table S1: Cohort Demographic Data

	Number (N = 48)	Percent
**Sex**		
Male	21	44
Female	27	56
**Age**		
1-17	8	17
18-54	32	67
55-70	8	17
**Race/Ethnicity**		
White	18	38
Asian	13	27
Hispanic/Latino	12	25
Black/African American	2	4
American Indian or Alaska Native	1	2
Pacific Islander/Native Hawaiian	1	2
Declined to Answer	1	2

Table S2: Individual target gene ddPCR operating parameters and primer and probe sequences

Target Gene	Primer Sequence	Cycling Conditions	Amplicon Size	Reference
SARS-CoV-2 N	Fw: CATTACGTTTGGTGGACCCT<br>Rv: CCTTGCCATGTTGAGTGAGA<br>Probe: CGCGATCAAAACAACGTCGG 5'FAM/ZEN/3'IBFQ	50C - 60min<br>95C - 5min<br>[95C - 30s<br>56C - 1min] x40<br>98C - 10min<br>4C - Hold	143 bp	Wolfe et al. 2021

SARS-CoV 2 ORF1a	Fw: CAGAACTGGAACCACCTTGT<br>Rv: TACAGTTGAATTGGCAGGCA<br>Probe: TGCCACAGTACGTCTACAAGC<br>5'HEX/ZEN/3'IBFQ	50C - 60min<br>95C - 5min<br>[95C - 30s<br>56C - 1min] x40<br>98C - 10min<br>4C - Hold	179 bp	Wolfe et al. 2021
BCoV	Fw: CTGGAAGTTGGTGGAGTT<br>Rv: ATTATCGGCCTAACATACATC<br>Probe: CCTTCATATCTATACACATCAAGTTGTT<br>5'FAM/ZEN/3'IBFQ	50C - 60min<br>95C - 5min<br>[95C - 30s<br>56C - 1min] x40<br>98C - 10min<br>4C - Hold	85 bp	Wolfe et al. 2021
PMMoV	Fw: GAGTGGTTTGACCTTAACGTTTGA<br>Rv: TTGTCGGTTGCAATGCAAGT<br>Probe: CCTACCGAAGCAAATG<br>5'HEX/ZEN/3'IBFQ	50C - 60min<br>95C - 5min<br>[95C - 30s<br>56C - 1min] x40<br>98C - 10min<br>4C - Hold	68 bp	Wolfe et al. 2021
CrAssphage CPQ064	Fw: TGTATAGATGCTGCTGCAACTGTACTC<br>Rv: CGTTGTTTTCATCTTTATCTTGTCCAT<br>Probe: CTGAAATTGTTCATAAGCAA<br>5'FAM/ZEN/3'IBFQ	95C - 5 min<br>[95C - 30s<br>56C - 1min] x40<br>98C - 10min<br>4C - Hold	126 bp	Stachler et al. 2017

Table S3: Estimates derived from logistic regression of SARS-CoV-2 fecal shedding prevalence for an outcome variable of log10(gc/mg-dw)

**Random Effects**					
**Groups**	**Name**	**Variance**	**Std. Dev**		
**ID**	(Intercept)	13.24	3.638		
**Fixed Effects**					
**Name**	**Type**	**Estimate**	**Std. Error**	**z-value**	**p-value**
**Intercept**		2.81747	0.94748	2.974	0.00294
**Day after symptom onset**	Continuous	-0.22399	0.03611	-6.204	5.52e-10
**Sex = Male**	Binary	0.14498	1.22669	0.118	0.90592

Table S4: Estimates derived from linear regression of concentrations of PMMoV and crAssphage fecal shedding for an outcome variable of log10(gc/mg-dw).

**crAssphage**						
**Random Effects**						
**Groups**	**Name**	**Variance**	**Std. Dev**			
**ID**	(Intercept)	4.6816	2.1637			
**Residual**		0.4219	0.6495			
**Fixed Effects**						
**Name**	**Type**	**Estimate**	**Std. Error**	**df**	**t-value**	**p-value**
**Intercept**		2.70163	0.44172	55.18068	6.116	1.03e-07
**Sex = Male**	Binary	-0.51143	0.63581	45.44786	-0.804	0.425
**Positive SARS-CoV-2 Fecal Shedding**	Binary	0.09748	0.10196	318.64308	0.956	0.340
**log10(PMMoV_conc**	Continuous	0.02513	0.02587	312.35886	0.971	0.332
**PMMoV**						
**Random Effects**						
**Groups**	**Name**	**Variance**	**Std. Dev**			
**ID**	(Intercept)	0.6787	0.8238			
**Residual**		2.0021	1.4149			
**Fixed Effects**						
**Name**	**Type**	**Estimate**	**Std. Error**	**df**	**t-value**	**p-value**

**Intercept**		4.78069	0.27345	64.19427	17.483	<2e-16
**Sex = Male**	Binary	-0.02717	0.29561	45.30766	-0.092	0.927
**Positive SARS-CoV-2 Fecal Shedding**	Binary	0.07691	0.19406	313.24624	0.396	0.692
**log10(crAss _conc**	Continuous	0.00974	0.06179	88.00628	0.158	0.875

Equation S1: Calculation converting the ddPCR generated concentration of gc/uL extract to gc/mg-dw.

$$\frac{Gene\ Copies}{mg\ dry\ weight\ stool} = \frac{Gene\ Copies}{\mu L\ Reaction} \times \frac{22\ \mu L\ Reaction}{5.5\ \mu L\ RNA\ Extract} \times \frac{100\ \mu L\ RNA\ Extract}{300\ \mu L\ Homogenate} \times \frac{1200\ \mu L\ Homogenate}{mg\ Stool} \times \frac{mg\ stool}{mg\ dry\ weight\ stool}$$

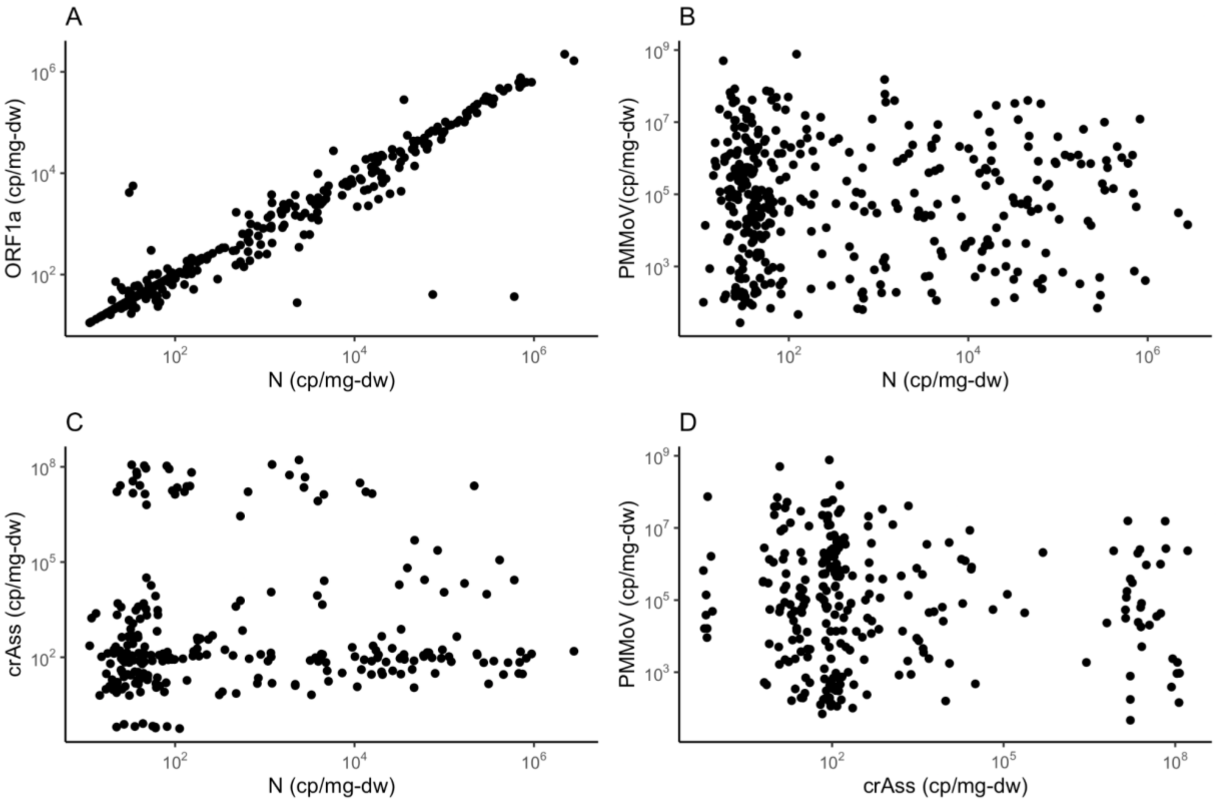

S1. Plots showing correlations between the different gene targets investigated in this study. A) SARS-CoV-2 N vs ORF1a, B) N vs PMMoV, C) N vs. crAss, D) crAss vs PMMoV

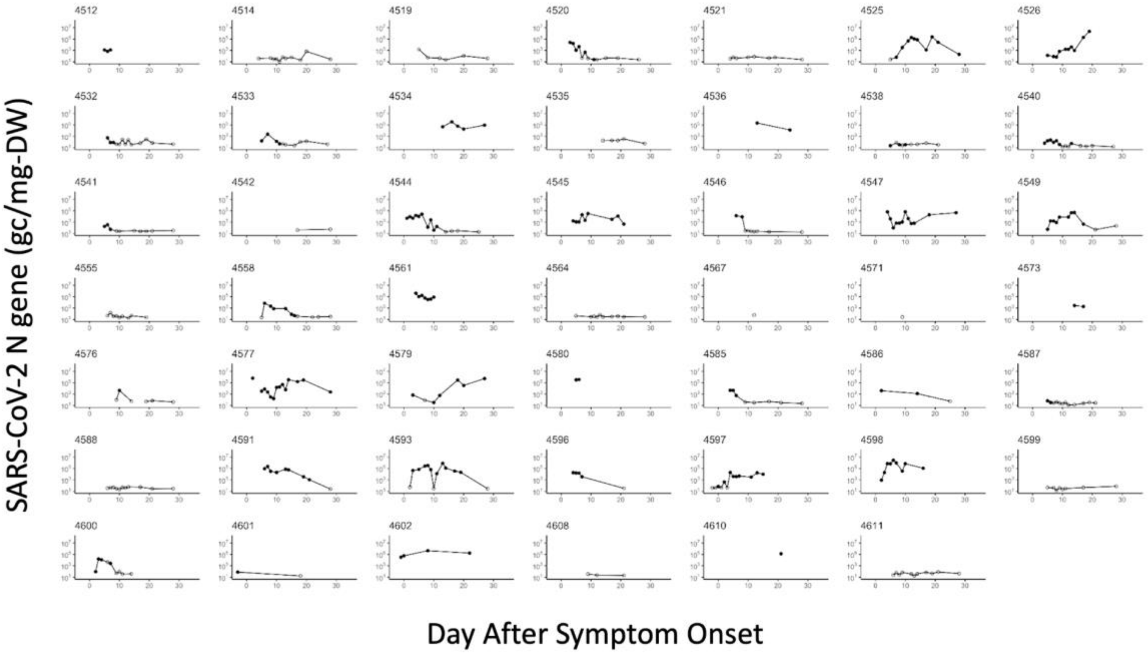

Figure S2. Individual SARS-CoV-2 Trajectories

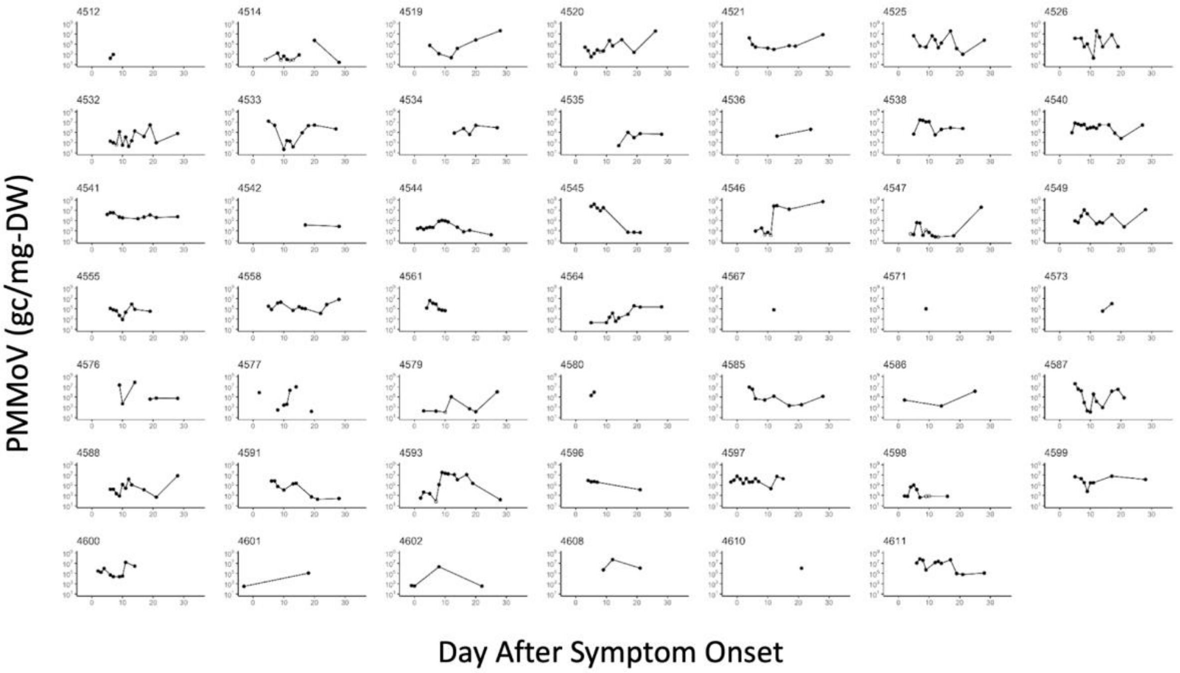

Figure S3: Individual PMMoV shedding trajectories

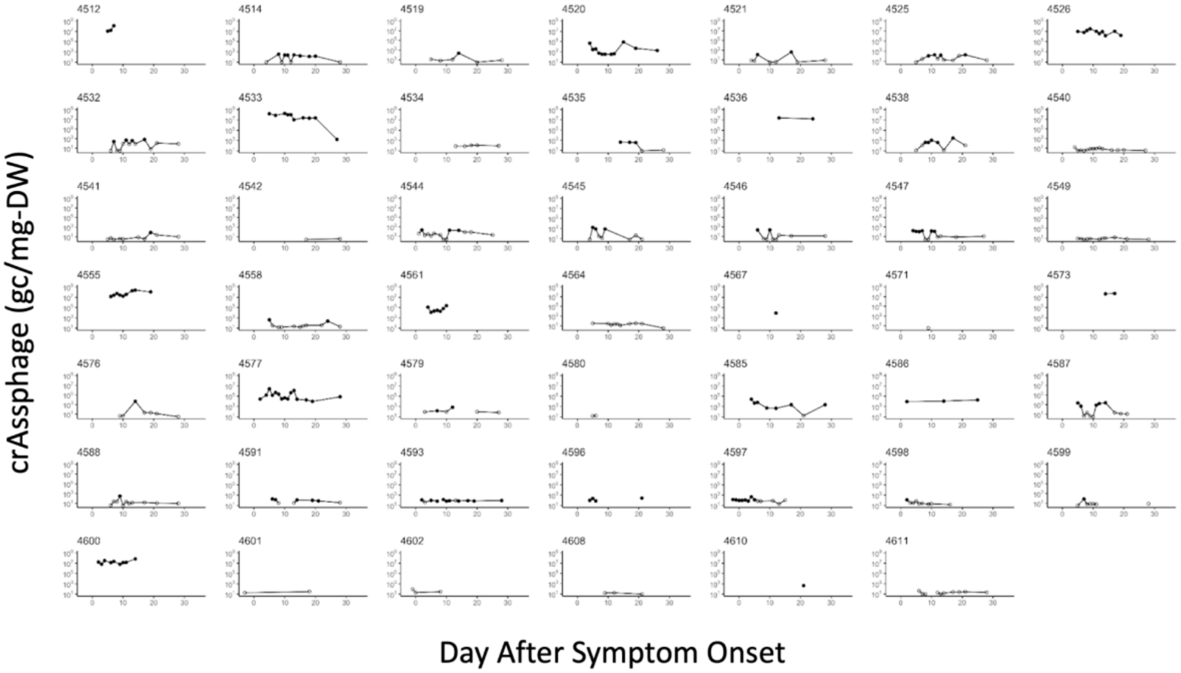

Figure S4. Individual crAssphage shedding trajectories

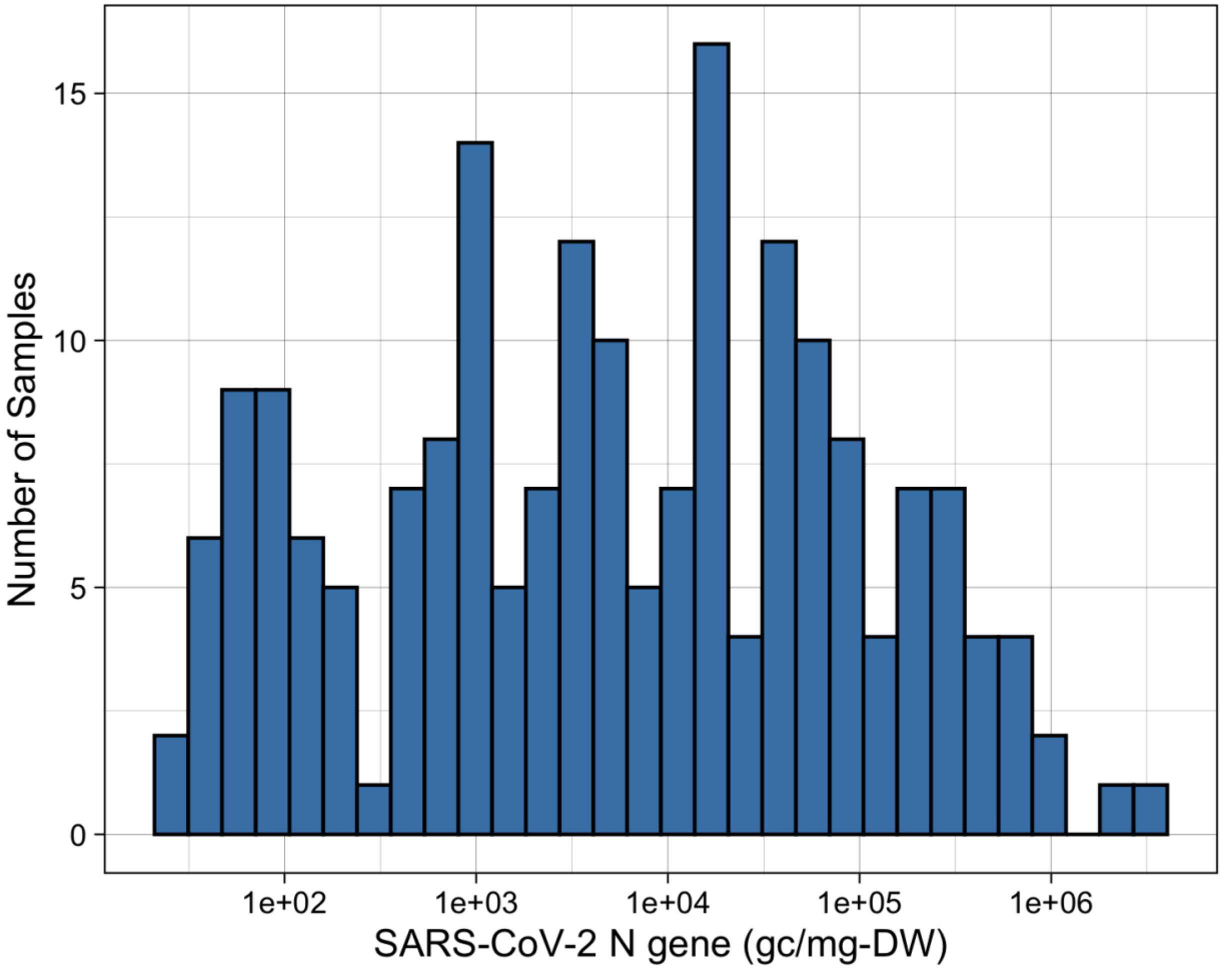

S5: Histogram showing the distribution of all above-LOB SARS-CoV-2 nucleocapsid gene measurements.

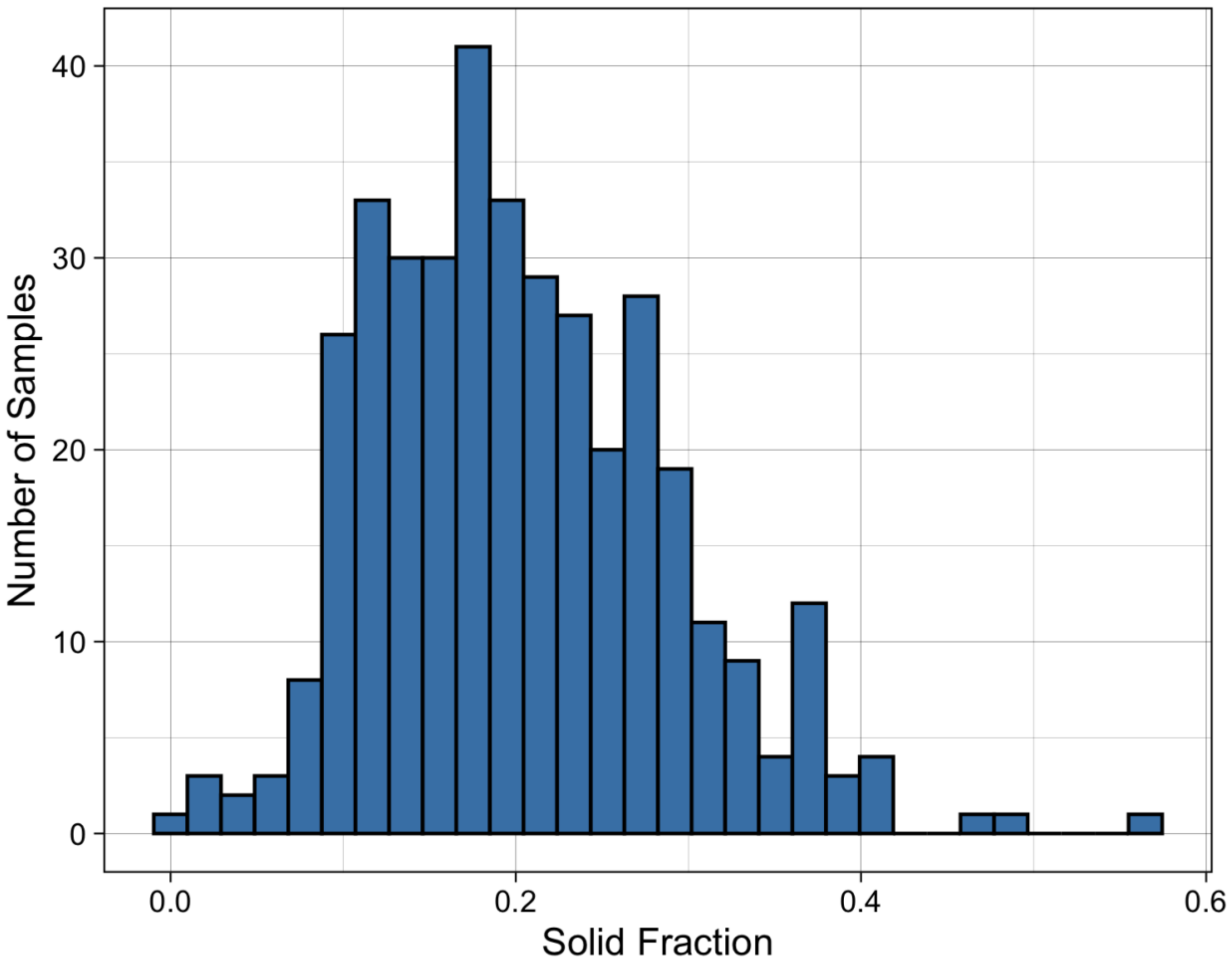

Figure S6: Histogram of the distribution of the solid fraction of all stool samples measured.

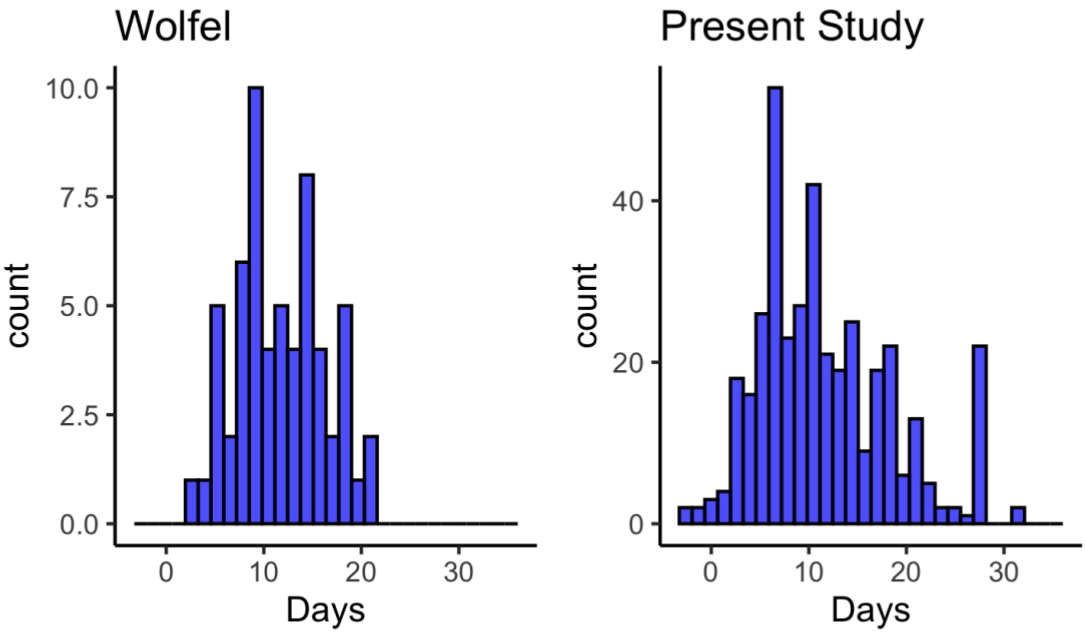

.

Figure S7: Histogram comparing the sampling coverage (post symptom onset) between Wölfel et al 2020, and the present study.

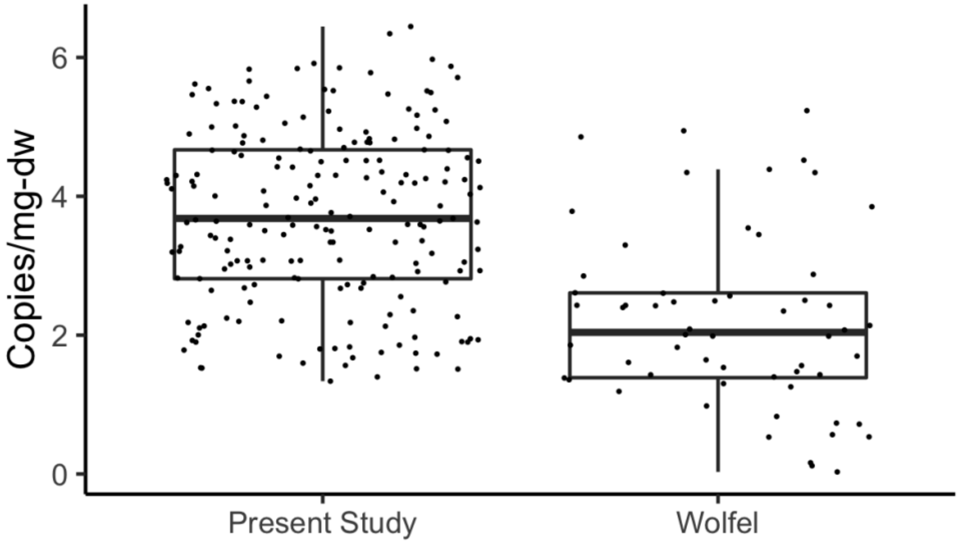

Figure S8: Comparison of the magnitudes of all positive measurements collected from this study with those collected in Wölfel et al 2020, assuming 20% dry mass samples.

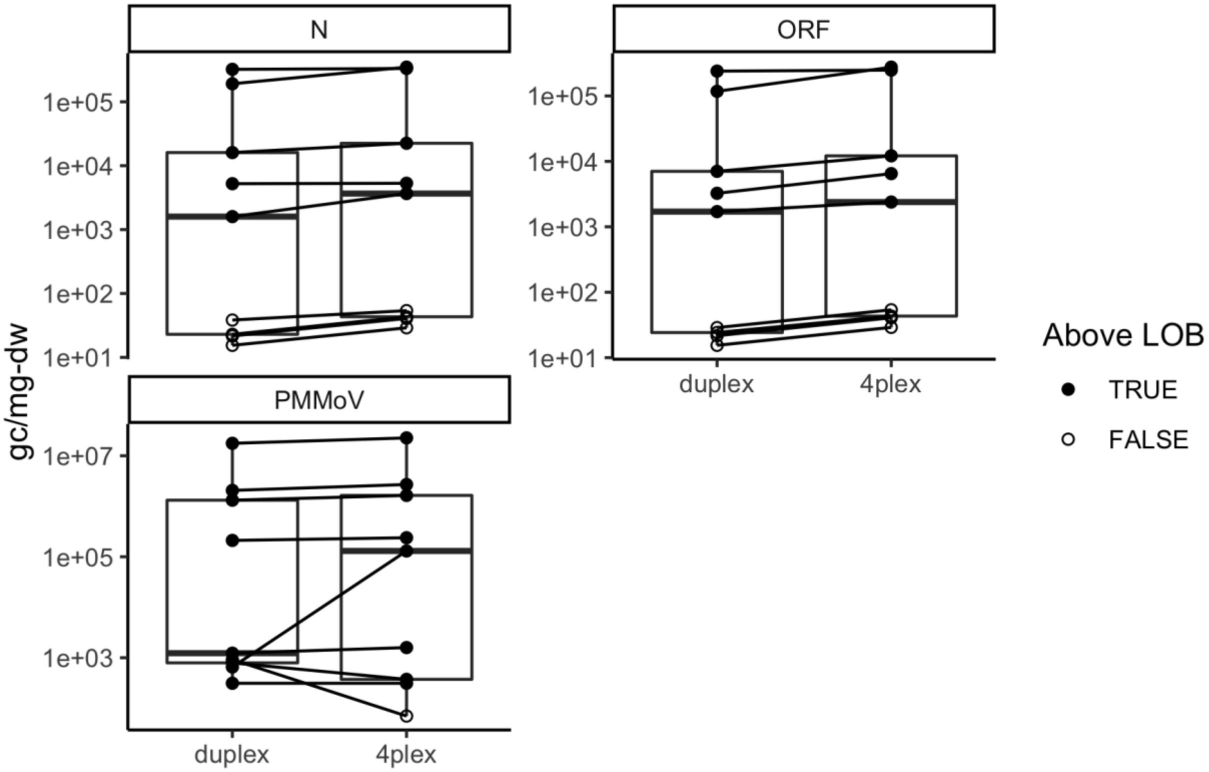

Figure S9: Side-by-side comparison of the RT-ddPCR 4plex assay with two duplex assays for the targets of interest.

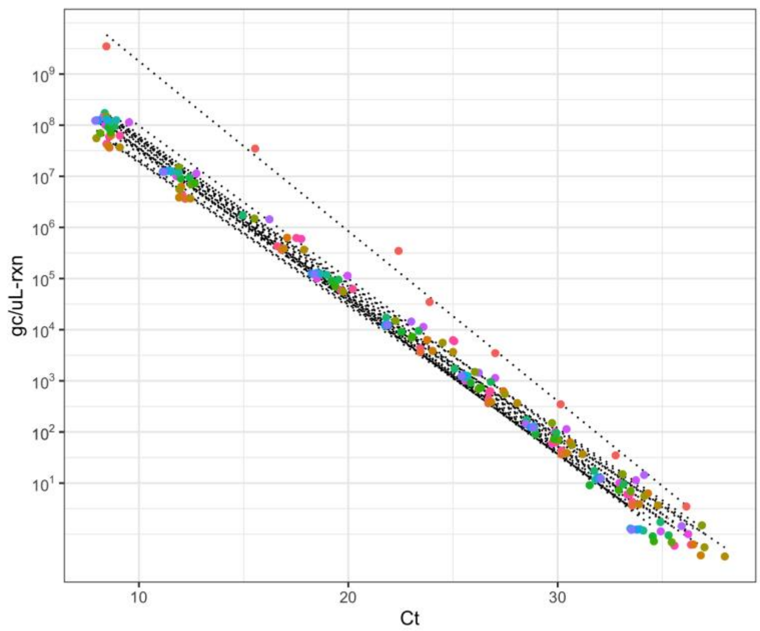

Figure S10: Standard curves correlating the Ct of crAssphage gene fragment dilutions as measured by qPCR to gene copies per microliter of reaction, measured by ddPCR.

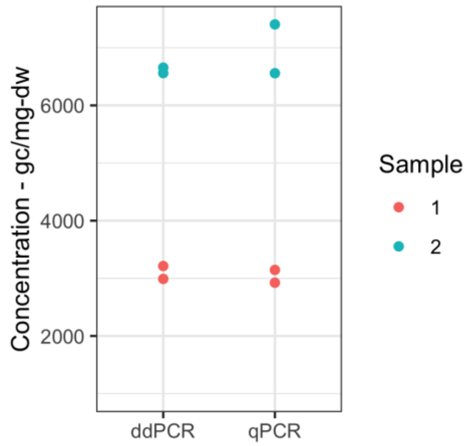

Figure S11: Comparison of quantified concentration of crAssphage positive stool samples from ddPCR vs qPCR with a ddPCR quantified standard curve. For each sample, extraction duplicates were measured using each method.

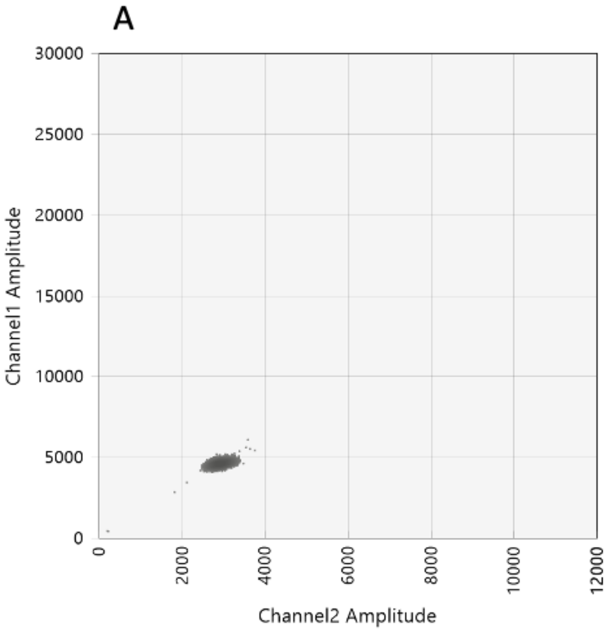

Figure S12: Examples of negative (A), and positive (B) samples measured by the 4 multiplexed ddPCR assay. The positive example exhibits a sample that was positive for all 4 targets.
